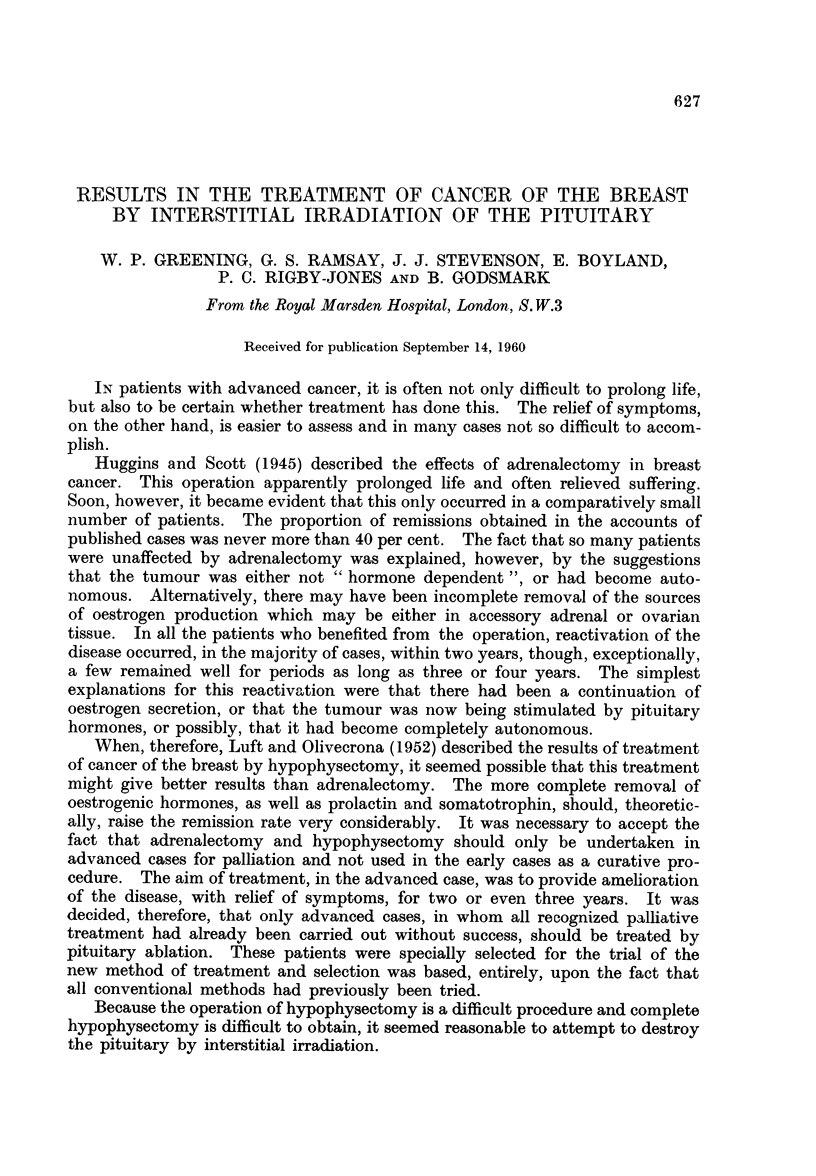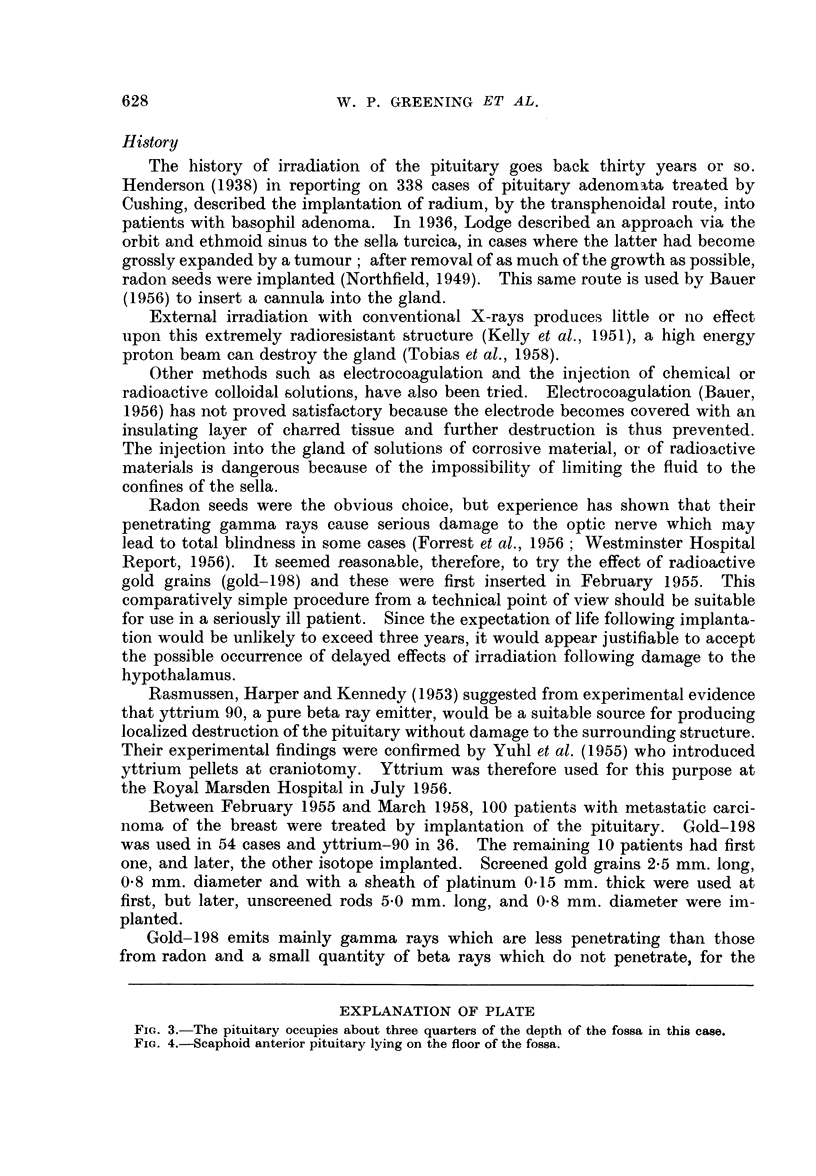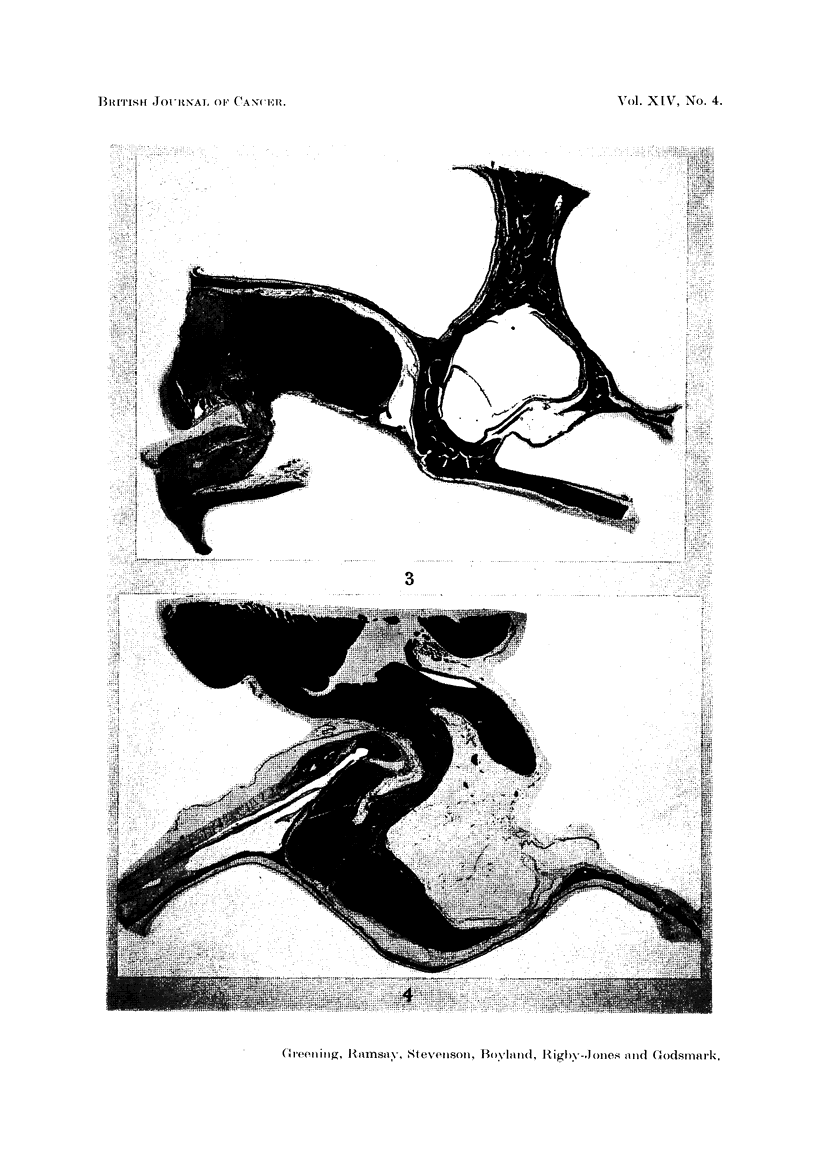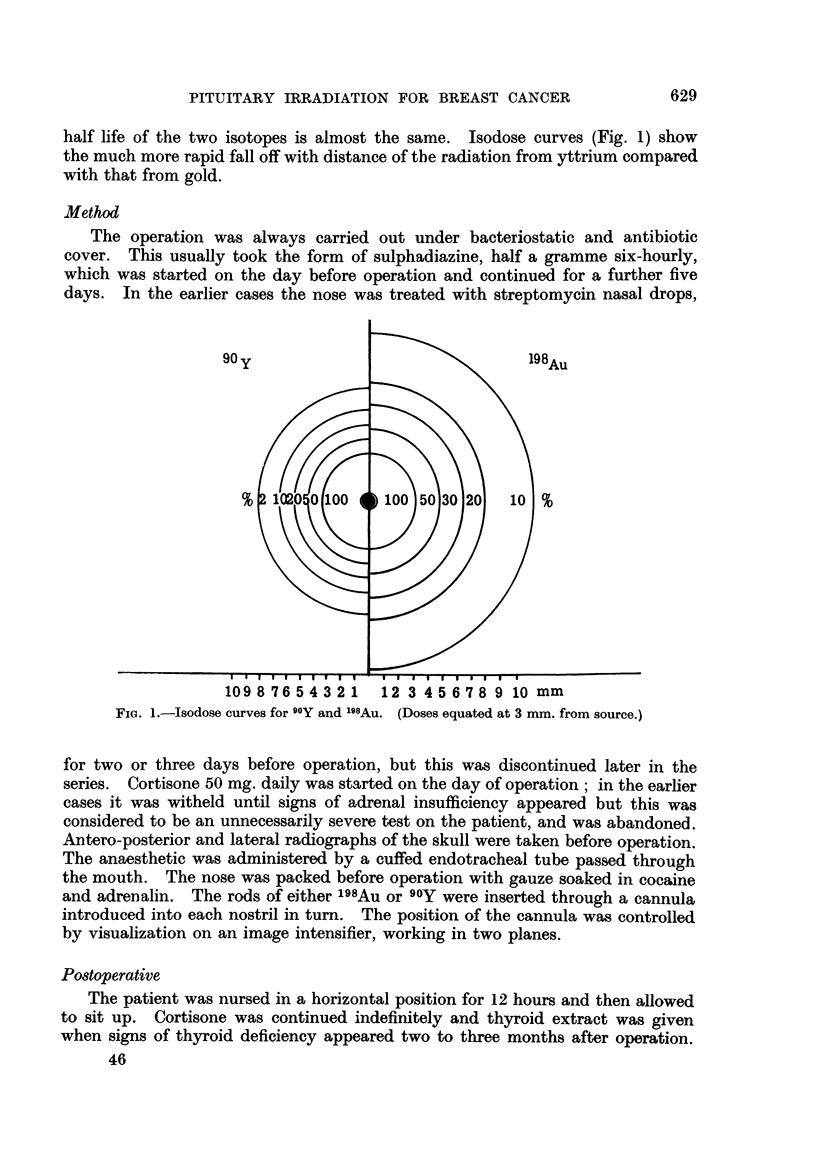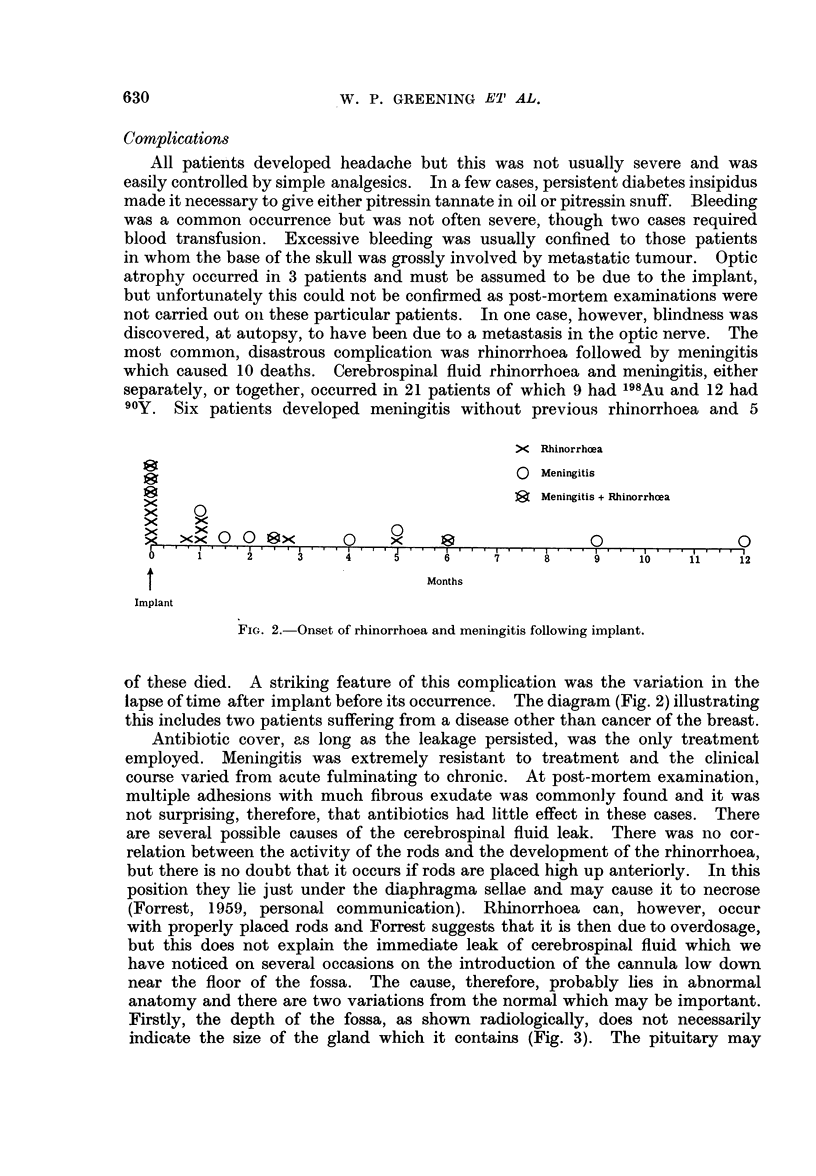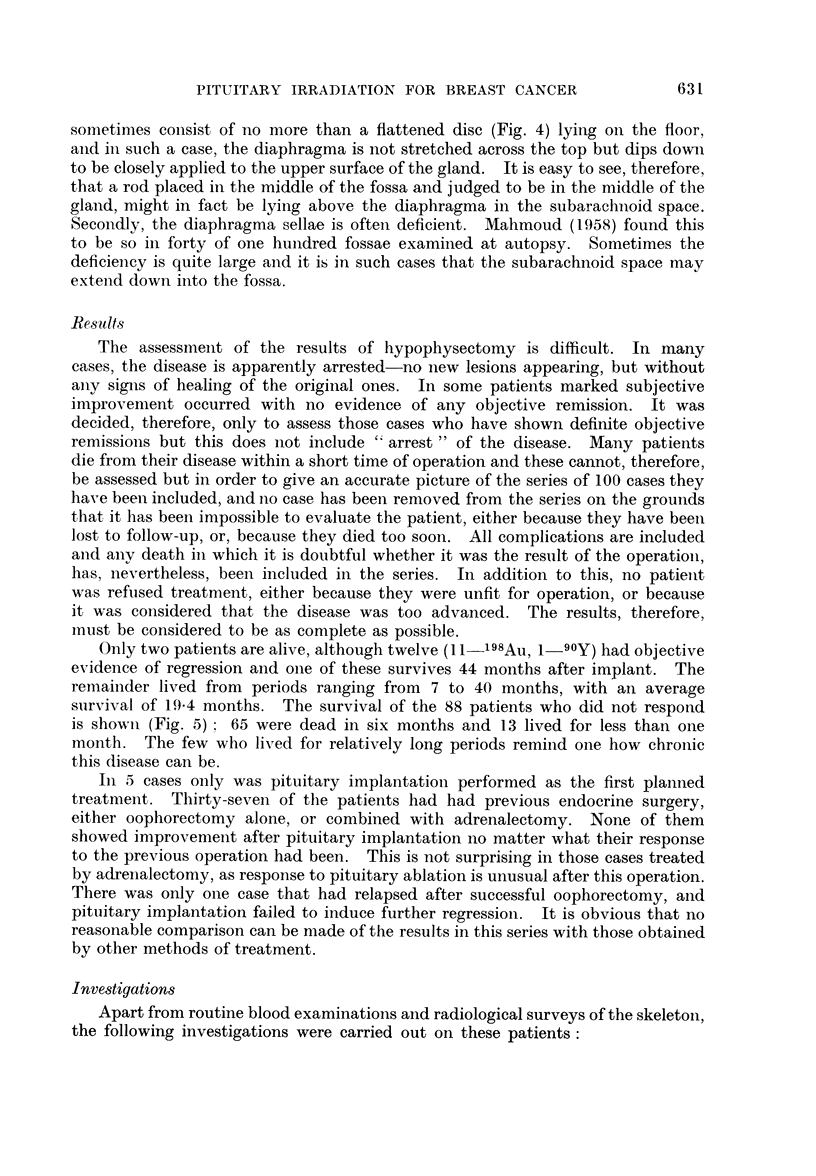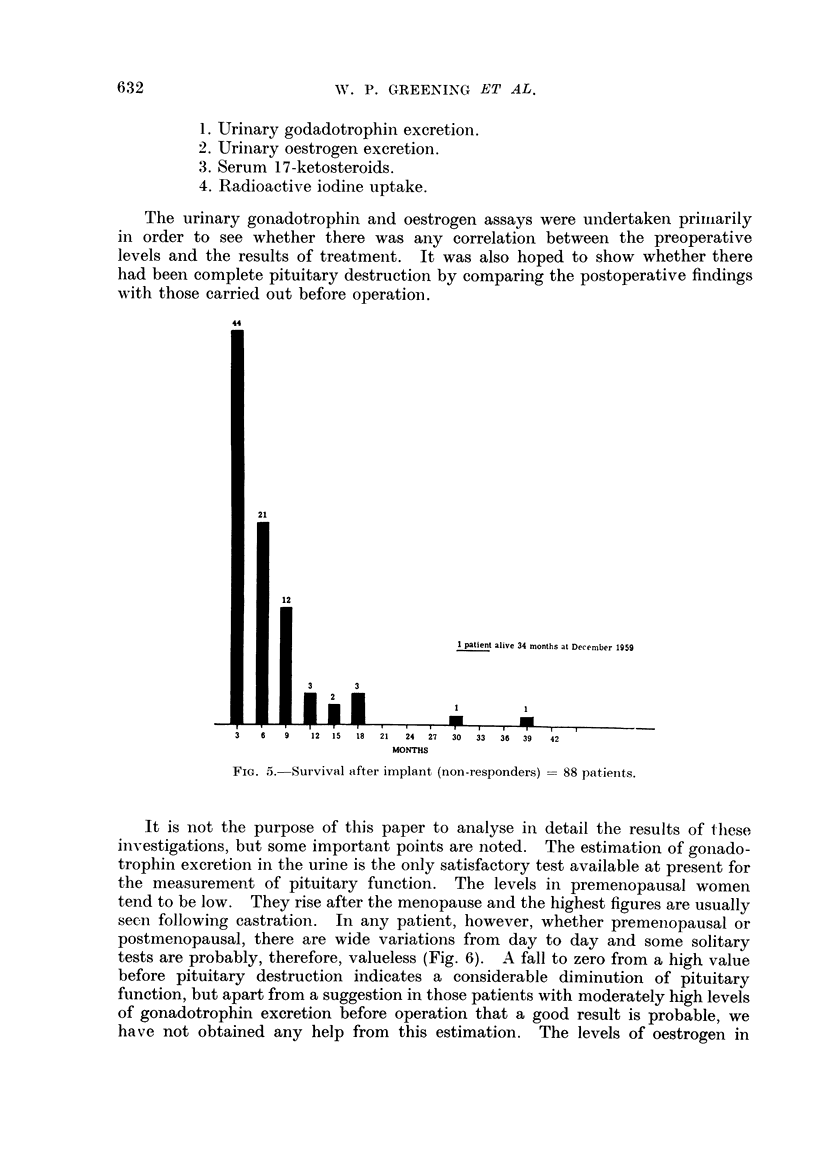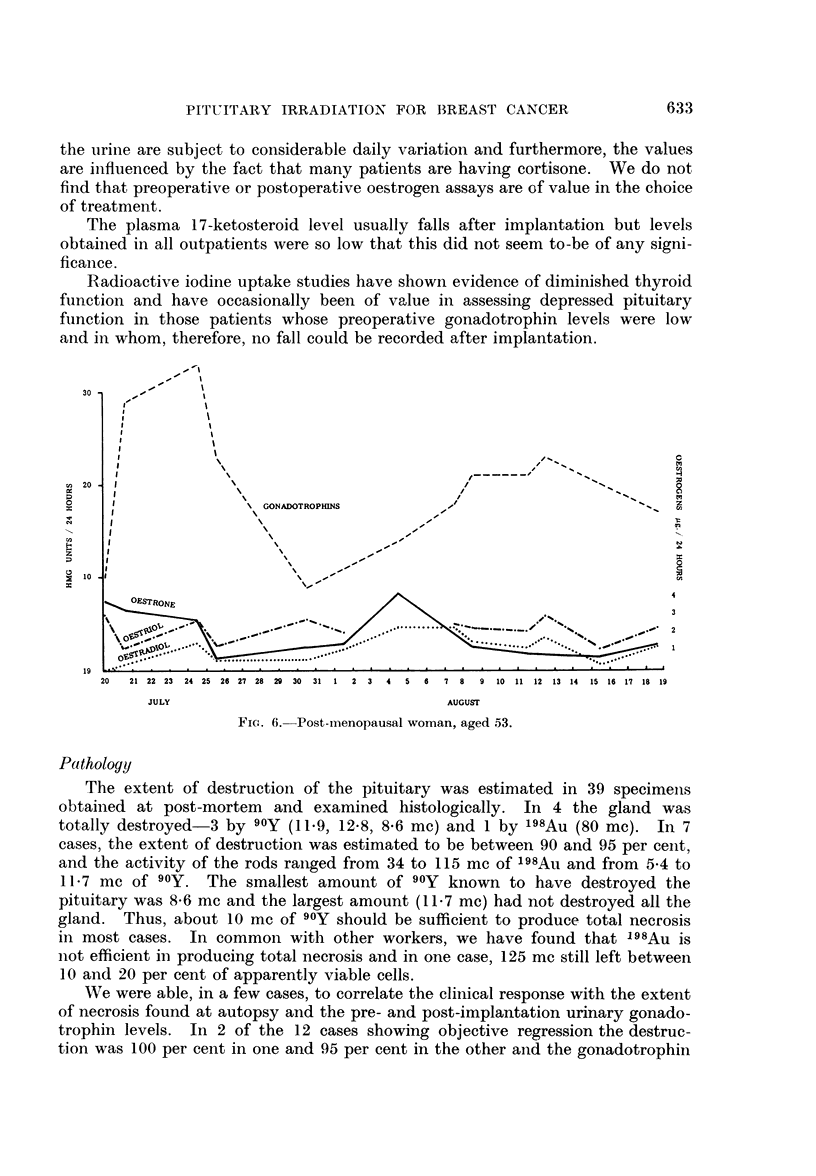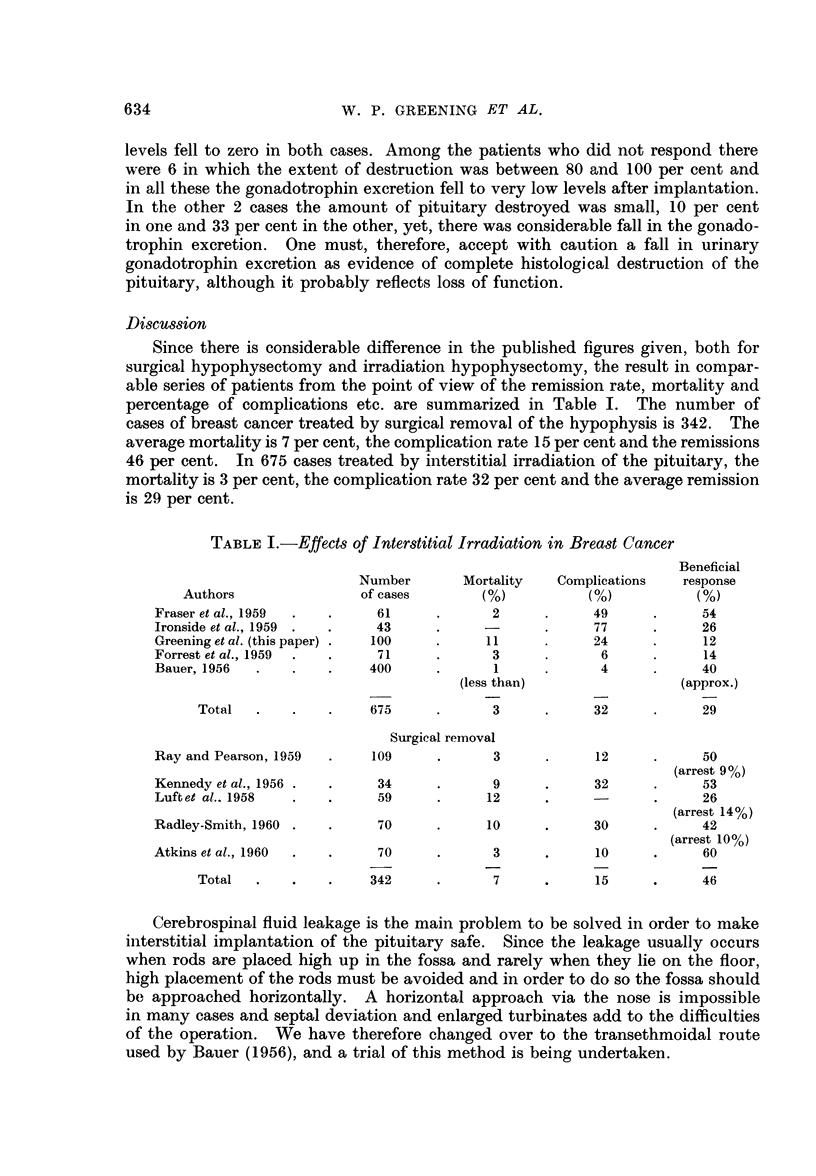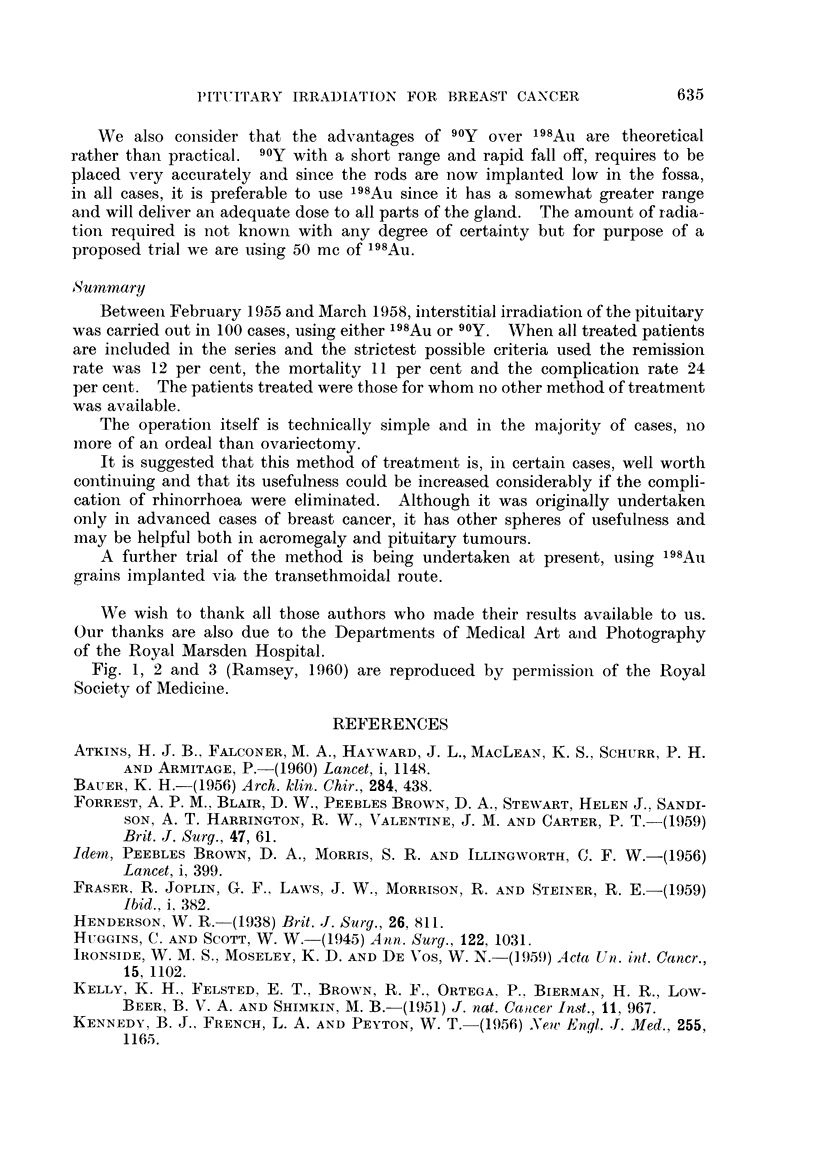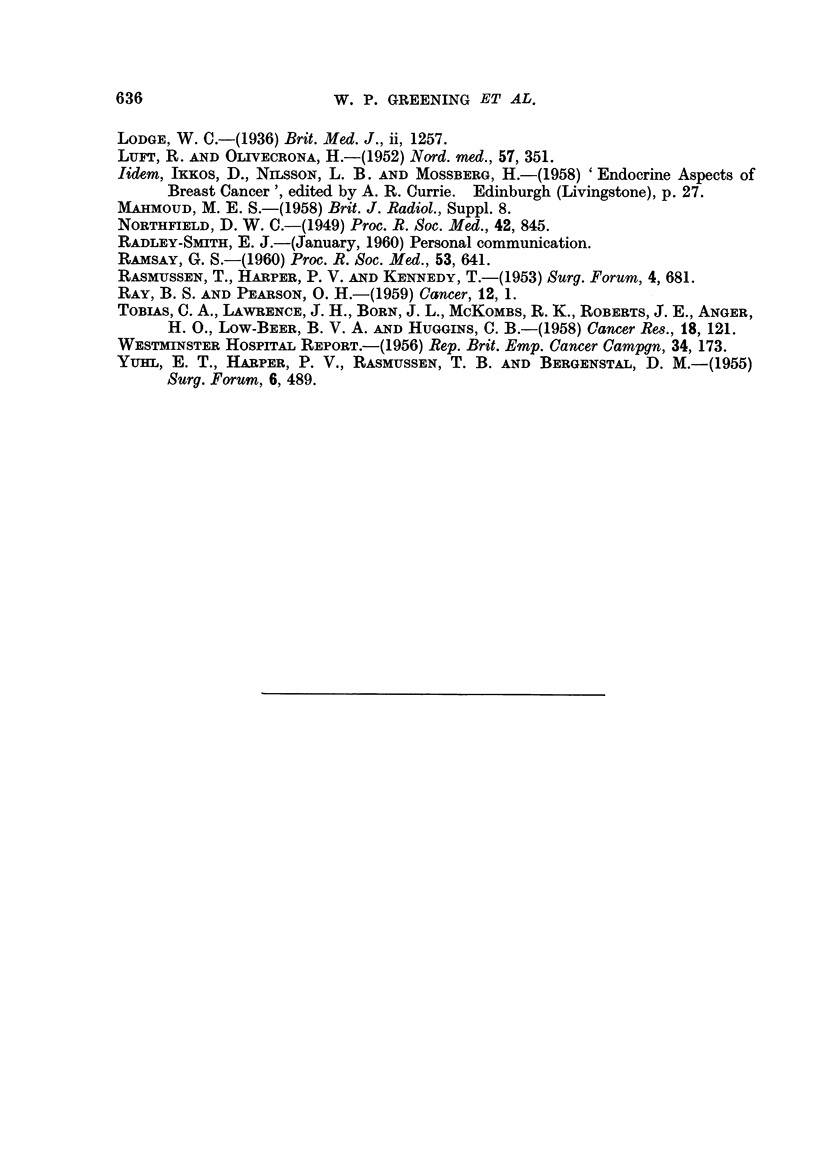# Results in the Treatment of Cancer of the Breast by Interstitial Irradiation of the Pituitary

**DOI:** 10.1038/bjc.1960.69

**Published:** 1960-12

**Authors:** W. P. Greening, G. S. Ramsay, J. J. Stevenson, E. Boyland, P. C. Rigby-Jones, B. Godsmark

## Abstract

**Images:**


### RESULTS IN THE TREATMENT OF CANCER OF THE BREAST BY INTERSTITIAL IRRADIATION OF THE PITUITARY

W. P. GREENING, G. S. RAMSAY, J. J. STEVENSON, E. BOYLAND, P. C. RIGBY-JONES AND B. GODSMARK

*From the Royal Marsden Hospital, London, S.W.3*

Received for publication September 14, 1960

IN patients with advanced cancer, it is often not only difficult to prolong life, but also to be certain whether treatment has done this. The relief of symptoms, on the other hand, is easier to assess and in many cases not so difficult to accomplish.

Huggins and Scott (1945) described the effects of adrenalectomy in breast cancer. This operation apparently prolonged life and often relieved suffering. Soon, however, it became evident that this only occurred in a comparatively small number of patients. The proportion of remissions obtained in the accounts of published cases was never more than 40 per cent. The fact that so many patients were unaffected by adrenalectomy was explained, however, by the suggestions that the tumour was either not " hormone dependent ", or had become autonomous. Alternatively, there may have been incomplete removal of the sources of oestrogen production which may be either in accessory adrenal or ovarian tissue. In all the patients who benefited from the operation, reactivation of the disease occurred, in the majority of cases, within two years, though, exceptionally, a few remained well for periods as long as three or four years. The simplest explanations for this reactivation were that there had been a continuation of oestrogen secretion, or that the tumour was now being stimulated by pituitary hormones, or possibly, that it had become completely autonomous.

When, therefore, Luft and Olivecrona (1952) described the results of treatment of cancer of the breast by hypophysectomy, it seemed possible that this treatment might give better results than adrenalectomy. The more complete removal of oestrogenic hormones, as well as prolactin and somatotrophin, should, theoretically, raise the remission rate very considerably. It was necessary to accept the fact that adrenalectomy and hypophysectomy should only be undertaken in advanced cases for palliation and not used in the early cases as a curative procedure. The aim of treatment, in the advanced case, was to provide amelioration of the disease, with relief of symptoms, for two or even three years. It was decided, therefore, that only advanced cases, in whom all recognized palliative treatment had already been carried out without success, should be treated by pituitary ablation. These patients were specially selected for the trial of the new method of treatment and selection was based, entirely, upon the fact that all conventional methods had previously been tried.

Because the operation of hypophysectomy is a difficult procedure and complete hypophysectomy is difficult to obtain, it seemed reasonable to attempt to destroy the pituitary by interstitial irradiation.

*History*

The history of irradiation of the pituitary goes back thirty years or so. Henderson (1938) in reporting on 338 cases of pituitary adenomata treated by Cushing, described the implantation of radium, by the transphenoidal route, into patients with basophil adenoma. In 1936, Lodge described an approach via the orbit and ethmoid sinus to the sella turcica, in cases where the latter had become grossly expanded by a tumour ; after removal of as much of the growth as possible, radon seeds were implanted (Northfield, 1949). This same route is used by Bauer (1956) to insert a cannula into the gland.

External irradiation with conventional X-rays produces little or no effect upon this extremely radioresistant structure (Kelly *et al.*, 1951), a high energy proton beam can destroy the gland (Tobias *et al.*, 1958).

Other methods such as electrocoagulation and the injection of chemical or radioactive colloidal solutions, have also been tried. Electrocoagulation (Bauer, 1956) has not proved satisfactory because the electrode becomes covered with an insulating layer of charred tissue and further destruction is thus prevented. The injection into the gland of solutions of corrosive material, or of radioactive materials is dangerous because of the impossibility of limiting the fluid to the confines of the sella.

Radon seeds were the obvious choice, but experience has shown that their penetrating gamma rays cause serious damage to the optic nerve which may lead to total blindness in some cases (Forrest *et al.*, 1956 ; Westminster Hospital Report, 1956). It seemed reasonable, therefore, to try the effect of radioactive gold grains (gold–198) and these were first inserted in February 1955. This comparatively simple procedure from a technical point of view should be suitable for use in a seriously ill patient. Since the expectation of life following implantation would be unlikely to exceed three years, it would appear justifiable to accept the possible occurrence of delayed effects of irradiation following damage to the hypothalamus.

Rasmussen, Harper and Kennedy (1953) suggested from experimental evidence that yttrium 90, a pure beta ray emitter, would be a suitable source for producing localized destruction of the pituitary without damage to the surrounding structure. Their experimental findings were confirmed by Yuhl *et al.* (1955) who introduced yttrium pellets at craniotomy. Yttrium was therefore used for this purpose at the Royal Marsden Hospital in July 1956.

Between February 1955 and March 1958, 100 patients with metastatic carcinoma of the breast were treated by implantation of the pituitary. Gold–198 was used in 54 cases and yttrium–90 in 36. The remaining 10 patients had first one, and later, the other isotope implanted. Screened gold grains 2·5 mm. long, 0·8 mm. diameter and with a sheath of platinum 0·15 mm. thick were used at first, but later, unscreened rods 5·0 mm. long, and 0·8 mm. diameter were implanted.

Gold–198 emits mainly gamma rays which are less penetrating than those from radon and a small quantity of beta rays which do not penetrate, for the

---

EXPLANATION OF PLATE

FIG. 3.—The pituitary occupies about three quarters of the depth of the fossa in this case.
FIG. 4.—Scaphoid anterior pituitary lying on the floor of the fossa.

3

4

Greening, Ramsay, Stevenson, Boyland, Rigby-Jones and Godsmark.

half life of the two isotopes is almost the same. Isodose curves (Fig. 1) show the much more rapid fall off with distance of the radiation from yttrium compared with that from gold.

*Method*

The operation was always carried out under bacteriostatic and antibiotic cover. This usually took the form of sulphadiazine, half a gramme six-hourly, which was started on the day before operation and continued for a further five days. In the earlier cases the nose was treated with streptomycin nasal drops,

FIG. 1.—Isodose curves for $^{90}Y$ and $^{198}Au$. (Doses equated at 3 mm. from source.)

for two or three days before operation, but this was discontinued later in the series. Cortisone 50 mg. daily was started on the day of operation ; in the earlier cases it was witheld until signs of adrenal insufficiency appeared but this was considered to be an unnecessarily severe test on the patient, and was abandoned. Antero-posterior and lateral radiographs of the skull were taken before operation. The anaesthetic was administered by a cuffed endotracheal tube passed through the mouth. The nose was packed before operation with gauze soaked in cocaine and adrenalin. The rods of either $^{198}Au$ or $^{90}Y$ were inserted through a cannula introduced into each nostril in turn. The position of the cannula was controlled by visualization on an image intensifier, working in two planes.

*Postoperative*

The patient was nursed in a horizontal position for 12 hours and then allowed to sit up. Cortisone was continued indefinitely and thyroid extract was given when signs of thyroid deficiency appeared two to three months after operation.

*Complications*

All patients developed headache but this was not usually severe and was easily controlled by simple analgesics. In a few cases, persistent diabetes insipidus made it necessary to give either pitressin tannate in oil or pitressin snuff. Bleeding was a common occurrence but was not often severe, though two cases required blood transfusion. Excessive bleeding was usually confined to those patients in whom the base of the skull was grossly involved by metastatic tumour. Optic atrophy occurred in 3 patients and must be assumed to be due to the implant, but unfortunately this could not be confirmed as post-mortem examinations were not carried out on these particular patients. In one case, however, blindness was discovered, at autopsy, to have been due to a metastasis in the optic nerve. The most common, disastrous complication was rhinorrhoea followed by meningitis which caused 10 deaths. Cerebrospinal fluid rhinorrhoea and meningitis, either separately, or together, occurred in 21 patients of which 9 had $^{198}Au$ and 12 had $^{90}Y$. Six patients developed meningitis without previous rhinorrhoea and 5

FIG. 2.—Onset of rhinorrhoea and meningitis following implant.

of these died. A striking feature of this complication was the variation in the lapse of time after implant before its occurrence. The diagram (Fig. 2) illustrating this includes two patients suffering from a disease other than cancer of the breast.

Antibiotic cover, as long as the leakage persisted, was the only treatment employed. Meningitis was extremely resistant to treatment and the clinical course varied from acute fulminating to chronic. At post-mortem examination, multiple adhesions with much fibrous exudate was commonly found and it was not surprising, therefore, that antibiotics had little effect in these cases. There are several possible causes of the cerebrospinal fluid leak. There was no correlation between the activity of the rods and the development of the rhinorrhoea, but there is no doubt that it occurs if rods are placed high up anteriorly. In this position they lie just under the diaphragma sellae and may cause it to necrose (Forrest, 1959, personal communication). Rhinorrhoea can, however, occur with properly placed rods and Forrest suggests that it is then due to overdosage, but this does not explain the immediate leak of cerebrospinal fluid which we have noticed on several occasions on the introduction of the cannula low down near the floor of the fossa. The cause, therefore, probably lies in abnormal anatomy and there are two variations from the normal which may be important. Firstly, the depth of the fossa, as shown radiologically, does not necessarily indicate the size of the gland which it contains (Fig. 3). The pituitary may sometimes consist of no more than a flattened disc (Fig. 4) lying on the floor, and in such a case, the diaphragma is not stretched across the top but dips down to be closely applied to the upper surface of the gland. It is easy to see, therefore, that a rod placed in the middle of the fossa and judged to be in the middle of the gland, might in fact be lying above the diaphragma in the subarachnoid space. Secondly, the diaphragma sellae is often deficient. Mahmoud (1958) found this to be so in forty of one hundred fossae examined at autopsy. Sometimes the deficiency is quite large and it is in such cases that the subarachnoid space may extend down into the fossa.

*Results*

The assessment of the results of hypophysectomy is difficult. In many cases, the disease is apparently arrested—no new lesions appearing, but without any signs of healing of the original ones. In some patients marked subjective improvement occurred with no evidence of any objective remission. It was decided, therefore, only to assess those cases who have shown definite objective remissions but this does not include " arrest " of the disease. Many patients die from their disease within a short time of operation and these cannot, therefore, be assessed but in order to give an accurate picture of the series of 100 cases they have been included, and no case has been removed from the series on the grounds that it has been impossible to evaluate the patient, either because they have been lost to follow-up, or, because they died too soon. All complications are included and any death in which it is doubtful whether it was the result of the operation, has, nevertheless, been included in the series. In addition to this, no patient was refused treatment, either because they were unfit for operation, or because it was considered that the disease was too advanced. The results, therefore, must be considered to be as complete as possible.

Only two patients are alive, although twelve (11—$^{198}$Au, 1—$^{90}$Y) had objective evidence of regression and one of these survives 44 months after implant. The remainder lived from periods ranging from 7 to 40 months, with an average survival of 19·4 months. The survival of the 88 patients who did not respond is shown (Fig. 5); 65 were dead in six months and 13 lived for less than one month. The few who lived for relatively long periods remind one how chronic this disease can be.

In 5 cases only was pituitary implantation performed as the first planned treatment. Thirty-seven of the patients had had previous endocrine surgery, either oophorectomy alone, or combined with adrenalectomy. None of them showed improvement after pituitary implantation no matter what their response to the previous operation had been. This is not surprising in those cases treated by adrenalectomy, as response to pituitary ablation is unusual after this operation. There was only one case that had relapsed after successful oophorectomy, and pituitary implantation failed to induce further regression. It is obvious that no reasonable comparison can be made of the results in this series with those obtained by other methods of treatment.

*Investigations*

Apart from routine blood examinations and radiological surveys of the skeleton, the following investigations were carried out on these patients:

1. Urinary godadotrophin excretion.
2. Urinary oestrogen excretion.
3. Serum 17-ketosteroids.
4. Radioactive iodine uptake.

The urinary gonadotrophin and oestrogen assays were undertaken primarily in order to see whether there was any correlation between the preoperative levels and the results of treatment. It was also hoped to show whether there had been complete pituitary destruction by comparing the postoperative findings with those carried out before operation.

FIG. 5.—Survival after implant (non-responders) = 88 patients.

It is not the purpose of this paper to analyse in detail the results of these investigations, but some important points are noted. The estimation of gonadotrophin excretion in the urine is the only satisfactory test available at present for the measurement of pituitary function. The levels in premenopausal women tend to be low. They rise after the menopause and the highest figures are usually seen following castration. In any patient, however, whether premenopausal or postmenopausal, there are wide variations from day to day and some solitary tests are probably, therefore, valueless (Fig. 6). A fall to zero from a high value before pituitary destruction indicates a considerable diminution of pituitary function, but apart from a suggestion in those patients with moderately high levels of gonadotrophin excretion before operation that a good result is probable, we have not obtained any help from this estimation. The levels of oestrogen in the urine are subject to considerable daily variation and furthermore, the values are influenced by the fact that many patients are having cortisone. We do not find that preoperative or postoperative oestrogen assays are of value in the choice of treatment.

The plasma 17-ketosteroid level usually falls after implantation but levels obtained in all outpatients were so low that this did not seem to-be of any significance.

Radioactive iodine uptake studies have shown evidence of diminished thyroid function and have occasionally been of value in assessing depressed pituitary function in those patients whose preoperative gonadotrophin levels were low and in whom, therefore, no fall could be recorded after implantation.

Fig. 6.—Post-menopausal woman, aged 53.

### *Pathology*

The extent of destruction of the pituitary was estimated in 39 specimens obtained at post-mortem and examined histologically. In 4 the gland was totally destroyed—3 by $^{90}$Y (11·9, 12·8, 8·6 mc) and 1 by $^{198}$Au (80 mc). In 7 cases, the extent of destruction was estimated to be between 90 and 95 per cent, and the activity of the rods ranged from 34 to 115 mc of $^{198}$Au and from 5·4 to 11·7 mc of $^{90}$Y. The smallest amount of $^{90}$Y known to have destroyed the pituitary was 8·6 mc and the largest amount (11·7 mc) had not destroyed all the gland. Thus, about 10 mc of $^{90}$Y should be sufficient to produce total necrosis in most cases. In common with other workers, we have found that $^{198}$Au is not efficient in producing total necrosis and in one case, 125 mc still left between 10 and 20 per cent of apparently viable cells.

We were able, in a few cases, to correlate the clinical response with the extent of necrosis found at autopsy and the pre- and post-implantation urinary gonadotrophin levels. In 2 of the 12 cases showing objective regression the destruction was 100 per cent in one and 95 per cent in the other and the gonadotrophin levels fell to zero in both cases. Among the patients who did not respond there were 6 in which the extent of destruction was between 80 and 100 per cent and in all these the gonadotrophin excretion fell to very low levels after implantation. In the other 2 cases the amount of pituitary destroyed was small, 10 per cent in one and 33 per cent in the other, yet, there was considerable fall in the gonadotrophin excretion. One must, therefore, accept with caution a fall in urinary gonadotrophin excretion as evidence of complete histological destruction of the pituitary, although it probably reflects loss of function.

*Discussion*

Since there is considerable difference in the published figures given, both for surgical hypophysectomy and irradiation hypophysectomy, the result in comparable series of patients from the point of view of the remission rate, mortality and percentage of complications etc. are summarized in Table I. The number of cases of breast cancer treated by surgical removal of the hypophysis is 342. The average mortality is 7 per cent, the complication rate 15 per cent and the remissions 46 per cent. In 675 cases treated by interstitial irradiation of the pituitary, the mortality is 3 per cent, the complication rate 32 per cent and the average remission is 29 per cent.

TABLE I.—*Effects of Interstitial Irradiation in Breast Cancer*

| Authors | Number of cases | Mortality (%) | Complications (%) | Beneficial response (%) |
|---|---|---|---|---|
| Fraser *et al.*, 1959 | 61 | 2 | 49 | 54 |
| Ironside *et al.*, 1959 | 43 | — | 77 | 26 |
| Greening *et al.* (this paper) | 100 | 11 | 24 | 12 |
| Forrest *et al.*, 1959 | 71 | 3 | 6 | 14 |
| Bauer, 1956 | 400 | 1 (less than) | 4 | 40 (approx.) |
| Total | 675 | 3 | 32 | 29 |
| | Surgical removal | | | |
| Ray and Pearson, 1959 | 109 | 3 | 12 | 50 (arrest 9%) |
| Kennedy *et al.*, 1956 | 34 | 9 | 32 | 53 |
| Luft *et al.*, 1958 | 59 | 12 | — | 26 (arrest 14%) |
| Radley-Smith, 1960 | 70 | 10 | 30 | 42 (arrest 10%) |
| Atkins *et al.*, 1960 | 70 | 3 | 10 | 60 |
| Total | 342 | 7 | 15 | 46 |

Cerebrospinal fluid leakage is the main problem to be solved in order to make interstitial implantation of the pituitary safe. Since the leakage usually occurs when rods are placed high up in the fossa and rarely when they lie on the floor, high placement of the rods must be avoided and in order to do so the fossa should be approached horizontally. A horizontal approach via the nose is impossible in many cases and septal deviation and enlarged turbinates add to the difficulties of the operation. We have therefore changed over to the transethmoidal route used by Bauer (1956), and a trial of this method is being undertaken.

We also consider that the advantages of $^{90}Y$ over $^{198}Au$ are theoretical rather than practical. $^{90}Y$ with a short range and rapid fall off, requires to be placed very accurately and since the rods are now implanted low in the fossa, in all cases, it is preferable to use $^{198}Au$ since it has a somewhat greater range and will deliver an adequate dose to all parts of the gland. The amount of radiation required is not known with any degree of certainty but for purpose of a proposed trial we are using 50 mc of $^{198}Au$.

*Summary*

Between February 1955 and March 1958, interstitial irradiation of the pituitary was carried out in 100 cases, using either $^{198}Au$ or $^{90}Y$. When all treated patients are included in the series and the strictest possible criteria used the remission rate was 12 per cent, the mortality 11 per cent and the complication rate 24 per cent. The patients treated were those for whom no other method of treatment was available.

The operation itself is technically simple and in the majority of cases, no more of an ordeal than ovariectomy.

It is suggested that this method of treatment is, in certain cases, well worth continuing and that its usefulness could be increased considerably if the complication of rhinorrhoea were eliminated. Although it was originally undertaken only in advanced cases of breast cancer, it has other spheres of usefulness and may be helpful both in acromegaly and pituitary tumours.

A further trial of the method is being undertaken at present, using $^{198}Au$ grains implanted via the transethmoidal route.

We wish to thank all those authors who made their results available to us. Our thanks are also due to the Departments of Medical Art and Photography of the Royal Marsden Hospital.

Fig. 1, 2 and 3 (Ramsey, 1960) are reproduced by permission of the Royal Society of Medicine.

### REFERENCES

ATKINS, H. J. B., FALCONER, M. A., HAYWARD, J. L., MACLEAN, K. S., SCHURR, P. H. AND ARMITAGE, P.—(1960) *Lancet*, i, 1148.

BAUER, K. H.—(1956) *Arch. klin. Chir.*, **284**, 438.

FORREST, A. P. M., BLAIR, D. W., PEEBLES BROWN, D. A., STEWART, HELEN J., SANDISON, A. T. HARRINGTON, R. W., VALENTINE, J. M. AND CARTER, P. T.—(1959) *Brit. J. Surg.*, **47**, 61.

*Idem*, PEEBLES BROWN, D. A., MORRIS, S. R. AND ILLINGWORTH, C. F. W.—(1956) *Lancet*, i, 399.

FRASER, R. JOPLIN, G. F., LAWS, J. W., MORRISON, R. AND STEINER, R. E.—(1959) *Ibid.*, i, 382.

HENDERSON, W. R.—(1938) *Brit. J. Surg.*, **26**, 811.

HUGGINS, C. AND SCOTT, W. W.—(1945) *Ann. Surg.*, **122**, 1031.

IRONSIDE, W. M. S., MOSELEY, K. D. AND DE VOS, W. N.—(1959) *Acta Un. int. Cancr.*, **15**, 1102.

KELLY, K. H., FELSTED, E. T., BROWN, R. F., ORTEGA, P., BIERMAN, H. R., LOW-BEER, B. V. A. AND SHIMKIN, M. B.—(1951) *J. nat. Cancer Inst.*, **11**, 967.

KENNEDY, B. J., FRENCH, L. A. AND PEYTON, W. T.—(1956) *New Engl. J. Med.*, **255**, 1165.

LODGE, W. C.—(1936) *Brit. Med. J.*, ii, 1257.
LUFT, R. AND OLIVECRONA, H.—(1952) *Nord. med.*, **57**, 351.
*Iidem*, IKKOS, D., NILSSON, L. B. AND MOSSBERG, H.—(1958) 'Endocrine Aspects of Breast Cancer', edited by A. R. Currie. Edinburgh (Livingstone), p. 27.
MAHMOUD, M. E. S.—(1958) *Brit. J. Radiol.*, Suppl. 8.
NORTHFIELD, D. W. C.—(1949) *Proc. R. Soc. Med.*, **42**, 845.
RADLEY-SMITH, E. J.—(January, 1960) Personal communication.
RAMSAY, G. S.—(1960) *Proc. R. Soc. Med.*, **53**, 641.
RASMUSSEN, T., HARPER, P. V. AND KENNEDY, T.—(1953) *Surg. Forum*, **4**, 681.
RAY, B. S. AND PEARSON, O. H.—(1959) *Cancer*, **12**, 1.
TOBIAS, C. A., LAWRENCE, J. H., BORN, J. L., McKOMBS, R. K., ROBERTS, J. E., ANGER, H. O., LOW-BEER, B. V. A. AND HUGGINS, C. B.—(1958) *Cancer Res.*, **18**, 121.
WESTMINSTER HOSPITAL REPORT.—(1956) *Rep. Brit. Emp. Cancer Campgn*, **34**, 173.
YUHL, E. T., HARPER, P. V., RASMUSSEN, T. B. AND BERGENSTAL, D. M.—(1955) *Surg. Forum*, **6**, 489.